# Population Genomics analysis of *Leptosphaeria biglobosa* Associated with *Brassica napus* in China Reveals That Geographical Distribution Influences Its Genetic Polymorphism

**DOI:** 10.3390/microorganisms12071347

**Published:** 2024-07-01

**Authors:** Yiji Shi, Zhiting Guo, Shunjun Bao, Jiali Xu, Keqi Li, Songbai Rong, Qiangsheng Li, Aixia Xu, Duojie Zhandui, Zhen Huang, Mingguang Chu

**Affiliations:** 1 Crop Research Institute, Anhui Academy of Agricultural Sciences, Hefei 230001, China; shiyj0718@nwafu.edu.cn (Y.S.); rong4213@sina.com (S.R.); shi_7771845@163.com (Q.L.); 2 State Key Laboratory of Crop Stress Biology for Arid Areas, College of Agronomy, Northwest A&F University, Xianyang 712100, China; 18003852705@163.com (Z.G.); 15664923669@163.com (S.B.); x18547522455@163.com (J.X.); likeqi1218@sina.com (K.L.); xuaixia@nwafu.edu.cn (A.X.); dj5136@sina.com (D.Z.); 3 Anhui Provincial Key Laboratory of Crop Quality Improvement, Hefei 230001, China

**Keywords:** *Leptosphaeria biglobosa*, genome, genetic diversity, population structure

## Abstract

Blackleg disease, a major threat to *Brassica* crops worldwide, is primarily caused by the pathogen *Leptosphaeria biglobosa*. Investigating the genetic variation of *L. biglobosa* is crucial for managing and preventing the disease in *Brassica napus*. To date, there is scarce genomic variation information available for populations of *L. biglobosa* in China. In this study, 73 *L. biglobosa* strains of canola stalks were collected from 12 provinces in China and subjected to re-sequencing. The 73 assemblies averaged 1340 contigs, 72,123 bp N50, and 30.17 Mb in size. In total, 9409 core orthogroups and 867 accessory orthogroups were identified. A total of 727,724 mutation loci were identified, including 695,230 SNPs and 32,494 indels. Principal component analysis (PCA) and population structure analysis showed that these strains could be divided into seven subgroups. The strains in most provinces were clustered into a single subgroup, suggesting a strong influence of the geographic environment on strain variation. The average nucleotide diversity (θπ) of all strains was 1.03 × 10^−3^, indicating important genetic diversity among strains from different regions of China. This study provides valuable resources for future comparative genomics, gives new insights into the population evolution of *L. biglobosa*, and supports the development of strategies for managing blackleg disease in canola.

## 1. Introduction

Blackleg, also known as Phoma stem canker, caused by *Leptosphaeria maculans* and/or *Leptosphaeria biglobosa*, is one of the most destructive diseases affecting cruciferous plants [1,2]. Particularly detrimental to rapeseed, blackleg has a broad host range and can infect a variety of plants. The disease perpetuates from one growing season to the next and can spread from one crop to another through infected rapeseed stalks. The cotyledon, true leaves, stems, and siliques of rapeseed are all likely to be infected by blackleg pathogens, resulting in seedling or adult death and reduced rapeseed yield and quality [3]. Rapeseed is extensively used for the production of edible vegetable oil, industrial oil, and biofuel, which ranks as the third-largest global contributor to vegetable oil production after palm and soybean [4]. The impact of this disease has been severe in various countries, including Australia [5], the United Kingdom [6], Canada [7], and France [8], leading to annual global production losses estimated at around USD 160 million [9,10].

By analyzing the genomes of 19 conserved proteins, researchers have estimated that *L. biglobosa* and *L. maculans* diverged approximately 22 million years ago [11,12]. In Europe, these two species often coexist on *Brassica* crops, although their population proportions vary across different countries [13]. *L. maculans*, known for its greater aggressiveness, often leads to severe epidemics and important yield losses [14,15]. Among these different species, *L. biglobosa* ‘brassicae’ is the most prevalent and has been identified in almost all rapeseed-growing regions. To date, while *L. biglobosa* remains the sole species identified in China, there are growing concerns regarding the potential introduction and spread of *P. lingam* [16]. The first report of *L. biglobosa* causing blackleg in China was on *Brassica campestris* ssp. in 2014 [1], followed by the identification of *L. biglobosa* ‘canadensis’ on *Brassica napus* [2] in 2021 and *L. biglobosa* ‘brassicae’ on *Brassica juncea* var. Multisecta in the same year [17]. Initially documented in Anhui, Hubei, and Guizhou Provinces [18], *L. biglobosa* has since been observed in all major oilseed rape producing areas of China [19].

The completion of the genome sequencing of three *Leptosphaeria* genomes (*L. maculans* JN3, *L. maculans* Nz-T4, and *L. biglobosa* G12-14) has paved the way for in-depth studies on regional characteristics of *L. biglobosa* [20]. While there have been investigations on the genetic origins of blackleg in China, research focusing on strain variation across different provinces remains limited [21]. To obtain a comprehensive understanding of the genetic variation and evolutionary relationships among *L. biglobosa* isolates in China, and to enhance the effective control of blackleg disease in oilseed rape, we conducted Next-Generation Sequencing (NGS) on 73 isolates sourced from across China. We analyzed genome variations, performed clustering, and explored evolutionary patterns among these isolates. This research enriches the genomic information available for *L. biglobosa*, providing deeper insights into their evolution within China. Simultaneously, it contributes to the improvement of management and control strategies for blackleg in oilseed rape.

## 2. Materials and Methods

### 2.1. Fungal Strains and DNA Extraction

In 2018, a total of 73 strains of *L. biglobosa* ‘brassica’ were collected from rapeseed exhibiting symptoms of blackleg in 12 provinces in China, including Anhui, Zhejiang, Henan, Sichuan, Jiangsu, Jiangxi, Shaanxi, Hunan, Inner Mongolia, Shanghai, Qinghai, and Hubei (Table S1). Diseased tissues were clipped from the samples and underwent surface disinfection. The tissues were disinfected with 75% alcohol for 1 min, followed by treatment with 2.5% NaClO for 2 min, and rinsed three times with sterile water. The disinfected tissues were then placed on potato dextrose agar (PDA) plates containing 0.5% lactic acid, with 4–5 tissues per plate, and incubated at 23 °C for 2–3 days in the dark. After mycelial growth was observed, a plug of mycelium from the colony edge was transferred to fresh PDA plates for purification. This process was repeated thrice to obtain pure strains, which were subsequently cultured on PDA and preserved at −80 °C in 50% glycerol.

### 2.2. Genome Sequencing

The CTAB method was employed to extract the DNA from each *L. biglobosa* ‘brassica’ strain. DNA quantification was performed using a fluorometer equipped with a fluorescent dye (Qubit3.0, Thermo Fisher Scientific, Waltham, MA, USA). Each DNA sample that met the quality standards was sent to the Majorbio company (Shanghai, China) for sequencing. The genome DNA was randomly fragmented to approximately 350 base pairs (bp) in length using ultrasonication. Sequencing adapters were then ligated to the fragments, followed by PCR amplification to enrich the adapter-ligated fragments. DNA sequencing was carried out on an Illumina NovaSeq6000 sequencing platform. The raw reads were filtered using fastp v0.23.4 [22] to remove low-quality reads.

### 2.3. Genome Assembly and Annotation

For genome assembly, SPAdes v3.5.0 [23] was used to assemble the reads of 73 strains. In the setup for SPAdes, the default values for k-mers were retained, and the MismatchCorrector module was integrated to address mismatches. Then, GapFiller v1.11 [24] was employed to fill gaps in the contigs obtained from the assembly, and finally, sequence correction was performed using PrInSeS-G v1.0.0 [25] to rectify any clipping errors and the insertion or deletion of small fragments during the assembly process.

The assembly was subjected to GeneMark v1.10 [26] for gene prediction, followed by the use of tRNAscan-SE v2.0.7 [27] for the identification of tRNA genes, RNAmmer v1.2 [28] for recognizing rRNA sequences. The assembled genome was analyzed using RepeatModeler-2.0.1 [29] to identify novel repeat sequences, and then RepeatMasker v4.1.1 [30] was applied to pinpoint and quantify the occurrence of these repeats throughout the genomic regions. We annotated the predicted coding genes using the NR (http://ncbi.nlm.nih.gov, accessed on 14 April 2023), GO (http://www.geeontology.org, accessed on 14 April 2023), and KEGG (http://www.Kegg.jp, accessed on 14 April 2023) databases.

According to the results of PCA and admixture, only one strain retained for presenting the groups with high homogeneity. The proteins of the selected strains were clustered, and their predicted proteins were assigned to orthologous groups of genes (orthogroups) using OrthoFinder v2.5.5 [31]. The core gene were defined as orthogroups accounting for 95% of the strains and accessory groups were defined as orthogroups accounting for less than 95% of the strains. Subsequently, EffectorP 3.0 [32] was used to predict the effectors for each orthogroups.

### 2.4. SNP Calling and Filtering

The clean reads were aligned to the reference genome GCA_022343325.1 [20] using bwa v0.7.17 [33] software. Samtools v1.9 [33] was employed to sort the alignment results and extract information such as sequencing depth and genome coverage for each sample. The Genome Analysis Toolkit (GATK) HaplotypeCaller engine (version 3.8-0) [34] was utilized for genotype calling. SNP variants were retained and filtered using the GATK VariantFiltration package with the following parameters: cluster-windowsize = 35, cluster-size = 3, FS ≤ 60, and QD ≥ 2.0. SNPs were annotated using SnpEff v5.0 [35], and their distribution on the chromosome was visualized using CMplot v4.5.1 [36]. Post-filtering for SNPs with a missing rate ≤ 20% and MAF ≥ 5% was performed using PLINK v1.9 [37], by which a total of 17,830 SNPs were obtained for further association analysis.

### 2.5. Population Structure and Principal Component Analysis

For computational efficiency, we pruned our dataset of variants that are in linkage using PLINK v1.9 [35] with the parameter indep-pairwise 50 10 0.2. Subsequently, the population structure of *L. biglobosa* ‘brassica’, based on genome SNPs, was assessed using the ADMIXTURE program (v1.3.0) [38]. For each number of populations (K) ranging from 1 to 10, ten runs were executed. The optimal K value was determined by the lowest cross-validation error (CV). Additionally, principal component analysis was conducted using PLINK [35] and visualized using the scatterplot3d package (v0.3-41) in R.

### 2.6. Phylogenetic Tree

Evolutionary distances between the strains were calculated using the maximum likelihood (ML) method by IQ-TREE v2.3.4 [39], which used the -m MFP option (ModelFinder) for model selection [40]. To measure branch support, 1000 bootstrap replicates were implemented. The phylogenetic tree was visualized using ggtree v3.12.0 [41].

### 2.7. Nucleotide Diversity and Fst

To explore the ties of consanguinity among the subpopulations, the nucleotide diversity (π) and fixation index (Fst) were calculated in sliding windows of 500 bp across the genome using VCFtools v0.1.16 [42]. The average pairwise divergence within a clade (θπ) was estimated for the entire genome among different clades. To estimate θπ for the whole genome, a sliding window of 500 bp with a 90% overlap between adjacent windows was utilized.

### 2.8. Linkage Disequilibrium

For linkage disequilibrium analysis, the average r^2^ value for each subgroup was computed over distance length (<1000 bp), and the decay of linkage disequilibrium was calculated and visualized using PopLDdecay v1.5 [43].

## 3. Results

### 3.1. Genome Assembly and Annotatiopn

In total, 73 *L. biglobosa* strains collected across 12 provinces in China were subjected to Illumina sequencing. Subsequently, genome assembly was performed on the genomes of these 73 strains, yielding 73 genome assemblies with sizes ranging from 28.53 to 34.18 Mb, averaging 30.17 Mb. The number of contigs was between 561 and 2874, with an average of 1340 contigs; N50 spanned 25,139 to 100,543 bp, with a mean of 72,123 bp. On average, repetitive sequences accounted for 5.32% of the genomes, with the lowest proportion observed in the strain SAX-2 at 1.72% and the highest in the strain SAX-1 at 11.46%. The predicted protein coding genes of each genome numbered from 9654 to 10,124, averaging 9398. Among these, an average of 9398 proteins were annotated against the NR database, 6652 against the GO database, and 2730 against the KEGG database (Table S1).

We employed OrthoFinder [31] to identify core and accessory orthogroups among the retained 35 strains (Table S2). A total of 10,276 orthogroups were identified, and 9409 (91.54%) classified as core orthogroups and 867 (8.46%) as accessory orthogroups. Among the 35 strains, AH-AAS-5 possessed the fewest accessory genes, with 292, while JX-2 had the most, with 403. Upon conducting effector prediction on all orthogroups, we detected a total of 4010 effectors, comprising 3681 cytoplasmic effectors and 329 apoplastic effectors. We found that 3091 effectors belonged to core orthogroups, constituting 32.85% of core genes, while 504 were associated with accessory genes, accounting for 58.13% of accessory genes. Evidently, accessory genes predicted a significantly higher number of effectors compared to core genes, suggesting substantial genetic variation in the pathogen’s adaptation to diverse environments.

### 3.2. Sequencing, SNP Calling, and Read Mapping

**Figure 1:**
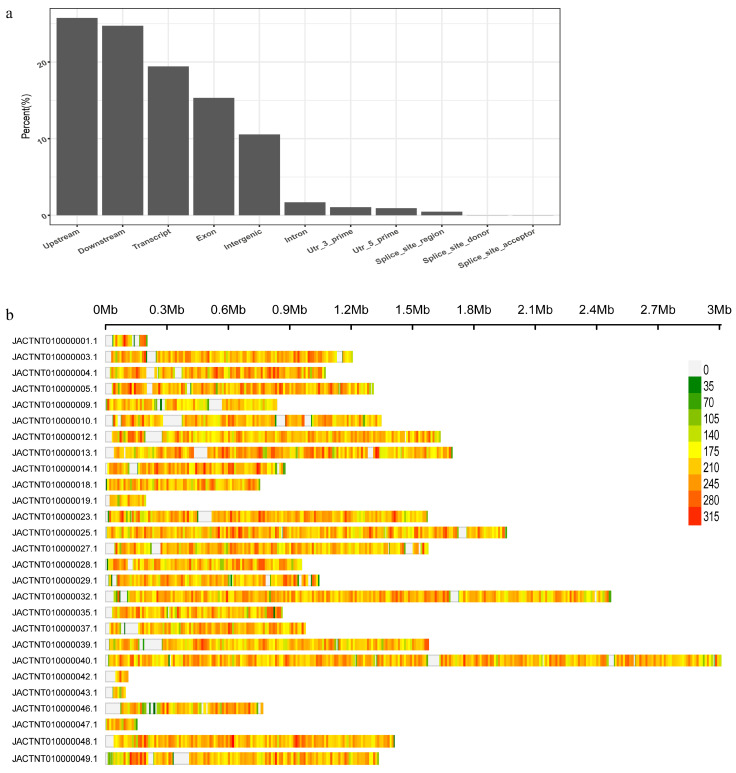
Characters of DNA variants of 70 strains of *Leptosphaeria biglobosa*. (**a**) The counts of variants annotation type by SnpEff. (**b**) The distribution of DNA variants in contigs with a 100 kb window size, shown only for contigs longer than 0.1 Mb in length.

In order to study the pathogenic characteristics, genetic diversity, and population structure of *L. biglobosa* ‘brassica’ strains in rapeseed in China and facilitate more effective control of blackleg, a total of 73 *L. biglobosa* strains were collected from 12 provinces in different regions of China. The whole genome of each of these 73 strains was resequenced and mapped to the reference genome [20]. It was found that the mapping rate of three samples was lower than 65%, meaning they were excluded from further analysis. The comparison rate of the remaining 70 strains was higher than 83%, with an average comparison rate of 92.34% and an average sequencing depth of 86× (Table S1). A total of 727,724 variant sites were identified by the GATK software (version 3.8-0), including 695,230 SNPs and 32,494 indels. The variant sites in the upstream and downstream of genes accounted for 25.76% and 24.74%, respectively, and the variants in exon accounted for 15.33% (Figure 1a). There were 108,853 variants causing non-synonymous mutations, of which 6558 were frame shift mutations. Subsequently, we investigated the distribution of SNPs across contigs and found that the distribution of SNPs was not uniform across or within contigs (Figure 1b). These findings highlight significant variations among *L. biglobosa* strains across different provinces, underscoring the importance of comprehensively studying strain variations in diverse regions to understand the full extent of genetic diversity of this pathogen.

### 3.3. Population Structure and Principal Component Analysis

**Figure 2:**
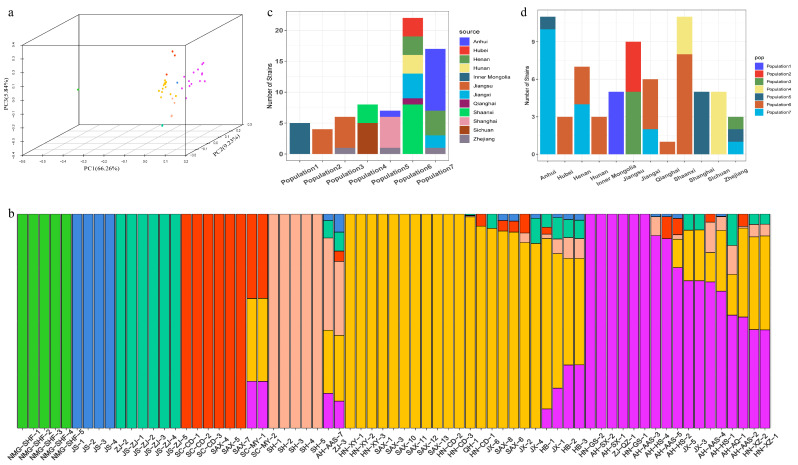
Population structure analysis of 69 *L. biglobosa* strains from China. (**a**) PCA analysis base SNPs of 69 strains. (**b**)The structure analysis of *L. biglobosa* populations in China K = 7. (**c**) Source statistics of strains from each population. (**d**) Classification of strains in each province.

The sample ZJ-1 was filtered out, because the SNP deletion rate was 24.88%. Finally, 69 strains were used for subsequent analysis. According to the PCA results, the 69 strains were divided into seven groups. The strains in Inner Mongolia were clustered together, exhibiting significantly different characteristics to strains from other regions. The contribution values of PC1, PC2, and PC3 were 66.26%, 9.22%, and 5.84%, respectively (Figure 2a). To analyze the population structure of these strains, ADMIXTURE was used for analysis. When K = 7, CV (the cross-validation error) reached the minimum, and the 69 strains were divided into seven subgroups (Figure 2b). All the strains from Inner Mongolia were clustered into Subgroup 1. The strains from Jiangsu Province were clustered into two subgroups, the strains from Nanjing were clustered into Subgroup 2, and the strains from Zhenjiang were clustered into Subgroup 3. All the strains from Sichuan province were clustered into Subgroup 4. However, the genetic diversity of two strains (SC-MY-1 and SC-MY-2) from Mianyang, Sichuan Province, was relatively high. All the strains from Shanghai were clustered into Subgroup 5. In addition, the strain AH-AAS-7 from Anhui and ZJ-3 from Zhejiang were also clustered into this subgroup and were highly heterozygous. There were 22 strains in Subgroup 6, including strains from six different provinces (Henan, Shaanxi, Qinghai, Hebei, Hunan, and Jiangxi). Except for AH-AAS-7, all strains from Anhui were clustered into Subgroup 7 (Figure 2b,c). The strains from Zhejiang, Henan, Shaanxi, Jiangxi, and Hunan were clustered into different subgroups (Figure 2d), indicating a high genetic variation within these regions. This diversity implies that the strains may have originated and spread from multiple regions, highlighting the complex dispersion patterns of *L. biglobosa* in these provinces.

### 3.4. Phylogenetic Tree

**Figure 3:**
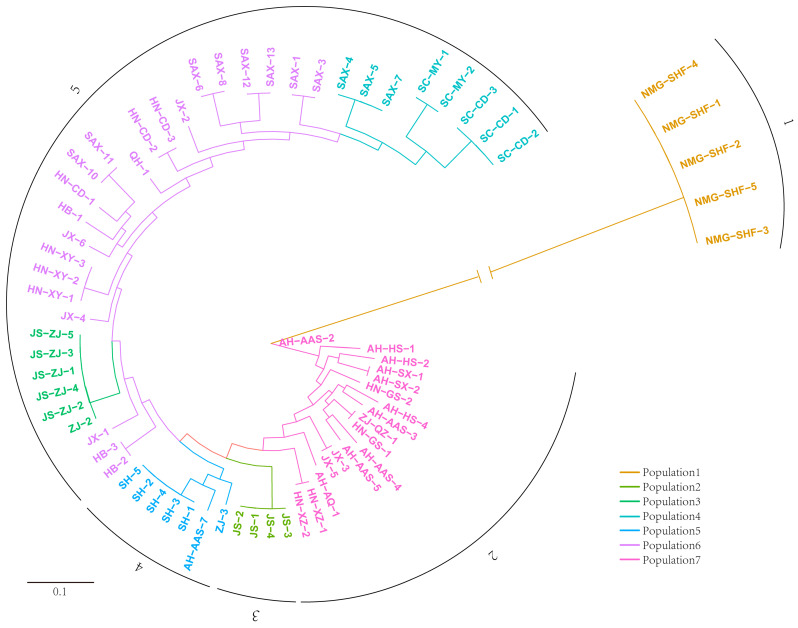
The phylogenetic tree of 69 strains from China was constructed using the maximum likelihood method according to SNP, different colors indicate different subgroups, and the 0.1 ruler in the bottom left corner indicates the branch length. The branch length of Population 1 was truncated and its value is 31.4. The phylogenetic tree is partitioned into five clades (labeled 1 to 5).

The phylogenetic trees of these 69 strains were constructed by iqtree, and the results were consistent with the results of population structure analysis. The five strains from Inner Mongolia were from one branch, and the length of the branch was the longest, reaching 31.4 (Figure 3). The results showed that the genetic variation of the strains from Inner Mongolia was significantly different from that of other regions, which is consistent with the PCA results. The main strains of Clade 2, corresponding to Subgroup 7, originated from Anhui, and a few strains were from Zhejiang, Jiangxi, Henan, and Hunan. Because Anhui borders Zhejiang, Jiangxi, and Henan Provinces, it is plausible that the strains from these regions were transmitted from Anhui (Figure 3). Clade 3 corresponds to Subgroup 2, which contains four strains, all of which were from Jiangsu. There are seven strains in Clade 4, five of which were from Shanghai. Clade 5 contains Subgroups 3, 4, and 6, with a total of 36 strains, which mainly came from Jiangsu, Sichuan, Shaanxi, Henan, and Hubei. The results suggest that the strains in these regions exhibit similar genetic variations, indicating potential inter-regional spread. Overall, the phylogeny of *L. biglobosa* strains appears to be influenced by geographic factors, likely due to local environmental stressors prompting mutations that enhance adaptation to specific regional conditions.

### 3.5. Genome Diversity

**Figure 4:**
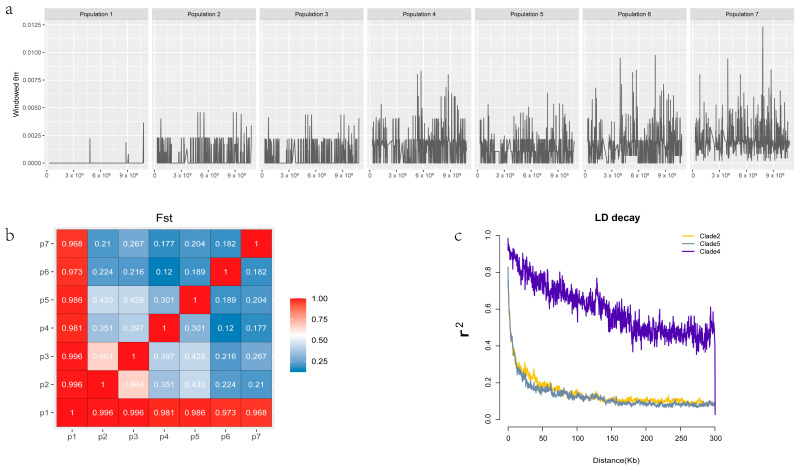
Genetic diversity of 69 *L. biglobosa* strains. (**a**) Nucleotide diversity (θπ) of 1 kb of chromosome sliding windows in different populations. (**b**) Group differentiation index (Fst) between different populations, p1 to p7 represent Population 1 to Population 7 individually. (**c**) Linkage disequilibrium (LD) decay in each clade, with Clade 1 and Clade 3 containing too few strains for LD decay to be calculated.

To determine the nucleic acid diversity among different subpopulations, genome-wide nucleotide diversity was evaluated by analyzing DNA sequences in order to quantify the extent of polymorphism at the nucleotide level. Nucleotide diversity reflects the average number of pairwise nucleotide differences among sequences and is influenced by the number of polymorphic sites and their respective frequencies. We used vcftools to calculate nucleotide diversity (θπ) with a sliding window size of 1 kb. The mean value (θπ) of these 69 strains was 1.03 × 10^−3^. The θπ of Clade 2 was the highest (1.99 × 10^−3^), followed by that of Clade 6 (1.74 × 10^−3^), and the θπ of Clade 1 (6.32 × 10^−6^) was the lowest. The 19 strains of Clade 2 were mainly from Anhui Province, indicating that Anhui Province had more bacterial variations. In contrast, Clade 1 only had four strains from Inner Mongolia. The limited sample size and geographically concentrated sampling in this clade led to a lower nucleotide diversity among these strains (Figure 4a).

The group differentiation index (F statistics, Fst) is considered to be a measure of genetic correlation between different subgroups in a stratified population. The closer the Fst value is to 1, the greater the degree of differentiation between subgroups, and the closer the Fst value is to 0, the smaller the degree of differentiation. The Fst values between Subgroup 1 and the other six subgroups were all close to 1 (Figure 4b), indicating that Subgroup 1 was far from the other subgroups. This result is consistent with the results of PCA and evolutionary trees, which further indicates that the strains from Inner Mongolia are genetically different to those from other parts of China. In addition to the large Fst between Subgroup 2 and Subgroup 3, the Fst between Subgroups 2, 3, 4, 5, 6, and 7 was relatively small, and the minimum Fst between Subgroup 4 and Subgroup 6 was 0.12. The results indicated that Subgroups 2, 3, 4, 5, 6, and 7 were closely related.

Linkage disequilibrium (LD) refers to the linkage relationship between two sites, which decreases with the increase in recombination and time. The LD attenuation of different populations is different, and slow attenuation indicates that the population has been selected. The LD decay was calculated using PopLDdecay, denoted by r^2^, and the distance to half of the maximum value was taken as the LD decay distance. According to the grouping results of these 69 strains from the evolutionary tree, the LD decay of each branch was calculated separately. Due to the small number and high similarity of strains contained in Clade 1 and 3, no results were obtained. The attenuation distances of Branch 2 and Branch 5 were 8.7 kb and 6.9 kb, respectively (Figure 4c). In contrast, the attenuation distance of Branch 4 was 118 kb. r^2^ fluctuated greatly, and the attenuation speed was slow, which might be due to the small number of strains in this branch.

## 4. Discussion

As a major canola producer, rapeseed cultivation in China spanned over 7.25 million hectares in 2022. Blackleg has reemerged as a significant challenge for global canola production [44,45]. At present, rapeseed in various provinces in China has been affected by blackleg. However, prevailing populations of the causal agent, *L. biglobosa*, are of low virulence, whereas *L. maculans* is absent in China [16]. Gaining insights into the relationship between the genetic diversity of the pathogen and its geographical distribution is instrumental for the effective monitoring and management of the disease. This study aims to examine the genome variations of *L. biglobosa* across various regions in China and to provide a theoretical basis for the prevention and control of blackleg in oilseed rape.

We performed genome assembly on the genomes of 73 strains of *L. biglobosa* with an average size of 30.17 Mb, 72,123 bp N50, and 1340 contigs; the reference sequence exhibited a size of 32.6 Mb, N50 of 1.4 Mb, and the number of contigs was 59. Although repetitive sequences are not directly transcribed into proteins like genes, they play important roles in gene expression, transcriptional regulation, and chromosome construction, affecting the evolution, inheritance, and variation of life [46,47]. Among the 73 assemblies, there were differences in repeat sequences, indicating that there were evolutionary differences among different strains, which may cause rich genetic diversity. The accessory genes in the population of *L. biglobosa* in China in this study accounted for 8.46% of the orthogroups, which was smaller than that of the proportion observed for other fungi (10–57%) [48], which may be caused by the insufficient genomic difference in the population. Interestingly, more effector ratios were predicted in accessory genes than in core genes, which also indicates the role of accessory genes in adapting to the environment.

We observed a relatively high degree of genomic variation among the collected isolates, which may be indicative of adaptation to their respective environments. In comparison to RAPD, ISSR, and SSR markers, fungal population diversity based on whole-genome resequencing is more reliable. In this study, we analyzed the genetic diversity, population structure, and inter-regional relationships of *L. biglobosa* ‘brassica’ collected in China by resequencing. Compared with the traditional markers, for instance, SSR, this method can obtain more abundant information about genetic variation.

To date, the genetic structure of blackleg pathogens in China, methods such as AFLP, ISSR-PCR, and SSR-PCR have been reported. In this study, NGS was used, providing a more comprehensive view of the genomic variations in blackleg pathogens. The results of the population structure analysis showed that the most appropriate number of subgroups of *L. biglobosa* in China was seven. This result is inconsistent with a previous study [45], in which *L. biglobosa* in winter oilseed rape was clustered into three subgroups using the SSR marker. The discrepancy between our findings and previous studies might stem from the limited scope of the latter, which focused solely on winter oilseed rape strains and employed a smaller set of SSR markers, potentially missing broader genetic variation. When considering whole-genome variations, 150 isolates from Canada were clustered into two main groups and six subgroups. However, when classified based on variations in *Avr* genes and small secreted protein-encoding genes, they fell into two main groups and four subgroups, indicating that utilizing whole-genome variation data yields more precise results [49].

The strains of Subgroup 7 are mainly distributed in the eastern coastal areas of China, including Anhui, Zhejiang, Jiangxi, and Henan. Subgroup 6, encompassing the most strains, is geographically west of Subgroup 7 and shows some overlap in strain sources, indicating that it could be the main pathogen type in China. The strains of Subgroup 4 mainly come from Shanghai and are found in the eastern coastal areas. The strains of Subgroups 2 and 3 are mainly from Jiangsu Province. In the phylogenetic tree analysis, Subgroups 3, 4, and 6 were clustered into the same group, indicating a close genetic distance among these three subgroups. Notably, the strains from Inner Mongolia cluster distinctly in a separate branch, with a significantly greater branch distance compared to other branches, potentially due to the region’s climate and the varieties cultivated there. These results indicate that the genetic diversity of *L. biglobosa* in different regions was affected by the geographical environment. The *L. biglobosa* strains in Jiangsu, Shaanxi, Jiangxi, and Hunan were divided into two subgroups (Figure 2d). This is in line with a prior study on 84 *L. biglobosa* strains in China [50], where ISSR markers were employed and their investigation revealed three main groups based on geographic origin, with Jiangsu Province strains being distributed across each group [51]. Zhuo et al. did not find that subgroups were divided by geographic location in China [45].

The analysis of the differentiation index among different subgroups revealed that, except for Subgroup 1, the other six subgroups exhibited minimal genetic disparity (Figure 4b). This variation might be attributed to the relatively recent emergence of blackleg in China [18], and the prevalence of a single pathogen ecotype leading to high genetic similarity among most subgroups. In practical agricultural settings, the circulation of seeds across different regions and sexual reproduction can lead to strains in various areas sharing similar genetic backgrounds. Fungi, such as the blackleg pathogen, enhance genetic exchange through mating, and the sexual reproduction of *L. maculans* can take place globally. This widespread exchange might result in reduced genetic differentiation within the European, Oceanic, and North American isolates [15]. The strains in Subgroup 1, originating from geographically remote Inner Mongolia, suggest that the strains might have undergone mutations unique to the local environmental conditions of this region.

## Data Availability

The datasets generated during the current study are available in the Sequence Read Archive (SRA) database of the National Center for Biotechnology Information (NCBI) under PRJNA1095415 accession.

## Supplementary Materials

The following supporting information can be downloaded at https://www.mdpi.com/article/10.3390/microorganisms12071347/s1, Table S1: The information and assembly statistics for field-collected *Leptosphaeria biglobosa* samples used in whole-genome sequencing. Mapping rate and average depth were calculated after mapping the reference genome; Table S2: The number of accessory orthogroups and predicted effectors in 35 strains.
